# Modulation of macrophage cytokine profiles during solid tumor progression: susceptibility to *Candida albicans *infection

**DOI:** 10.1186/1471-2334-9-98

**Published:** 2009-06-17

**Authors:** Marcela R Camargo, James Venturini, Fátima R Vilani-Moreno, Maria Sueli P Arruda

**Affiliations:** 1 Experimental Immunopathology Laboratory, Department of Biological Sciences, College of Sciences, São Paulo State University, UNESP, Bauru, SP, 17047-001, Brazil; 2 Botucatu Medical School, São Paulo State University, UNESP, Botucatu, SP, 18618-970, Brazil; 3 Instituto Lauro de Souza Lima, Bauru, SP, 17034-971, Brazil

## Abstract

**Background:**

In order to attain a better understanding of the interactions between opportunist fungi and their hosts, we investigated the cytokine profile associated with the inflammatory response to *Candida albicans *infection in mice with solid Ehrlich tumors of different degrees.

**Methods:**

Groups of eight animals were inoculated intraperitoneally with 5 × 10^6 ^*C. albicans *7, 14 or 21 days after tumor implantation. After 24 or 72 hours, the animals were euthanized and intraperitoneal lavage fluid was collected. Peritoneal macrophages were cultivated and the levels of IFN-γ, TNF-α, IL-12, IL-10 and IL-4 released into the supernatants were measured by ELISA. Kidney, liver and spleen samples were evaluated for fungal dissemination. Tumor-free animals and animals that had only been subjected to *C. albicans *infection were used as control groups.

**Results:**

Our results demonstrated that the mice produced more IFN-γ and TNF-α and less IL-10, and also exhibited fungal clearance, at the beginning of tumor evolution. With the tumor progression, this picture changed: IL-10 production increased and IFN-γ and TNF-α release decreased; furthermore, there was extensive fungal dissemination.

**Conclusion:**

Our results indicate that solid tumors can affect the production of macrophage cytokines and, in consequence, affect host resistance to opportunistic infections.

## Background

Systemic *Candida albicans *infections have a marked impact on the clinical course and outcome of cancer patients [1,2]; they are responsible for prolonged hospital stays, high healthcare costs and significant mortality [3-6]. Although a compromised immune response has been invoked as a cause of susceptibility to major infections in tumor-bearing patients, some authors disagree, arguing that the tumoral condition may, *inter alia*, result in active protection against opportunist pathogens [7]. As most knowledge about *Candida *infections in cancer patients has been obtained from patients with hematological malignancies [8], and since candidemia is an important complication in patients with solid tumors, we decided to study this premise using peritoneal macrophages in mice bearing solid Ehrlich tumors and infected with *C. albicans*. Previous studies have suggested an important role for macrophages in the control of candidiasis [9,10]. Those cells recognizably have a critical role in innate immunity and in the polarization of the immune adaptive response. They express complex functions including the production of cytokines that modulate the responses of other immune system cells and themselves as well, and bactericidal/tumoricidal activities [8]. Thus, if macrophage activities are compromised, the host may become more susceptible to infections and tumors and this may prejudice the results of specific treatments. The peritoneal macrophage population was chosen on the basis of the report of Bhaumik *et al. *[11] about macrophage traffic between the peritoneum and neoplastic tissues. According to those authors, during the evolution of the neoplasm, macrophages originating from the tumor will lodge within the peritoneal cavity.

Numerous studies have described a significant decrease in macrophage functions in tumor-bearing hosts [7]. Since we found no account in the literature about the role of cytokines in the development of solid tumors, and since there are divergent views about the evolution and gravity of *C. albicans *infection under such circumstances, we proposed to investigate those questions, evaluating the kinetics of cytokine production in mice that bore solid Ehrlich tumors (BTM) and were infected or not infected with the fungus.

## Methods

### Animals

Male Swiss mice (two months old) from the Animal House of São Paulo State University, Botucatu, SP, Brazil, were housed in groups of 3–5 animals and were provided with food and water *ad libitum*. All the protocols accorded with the ethical principles for animal research adopted by the Brazilian College of Animal Experimentation (COBEA). This study was approved by Ethical Committee of School of Sciences, Sao Paulo State University.

### Experimental design

The mice were divided into four groups: two BTM groups inoculated or not inoculated with *C. albicans *(Ca-BTM and BTM, respectively); and two control groups, tumor-free mice that were inoculated or not inoculated with *C. albicans *(Ca-CTL and CTL, respectively). The BTM group animals were killed on days 7, 14 or 21 after tumor implantation. The Ca-BTM group animals were inoculated intraperitoneally (i.p.) with *C. albicans *(5 × 10^6 ^fungi) on days 7, 14 or 21 after tumor implantation and were killed 24 or 72 h after *C. albicans *inoculation. Finally, the Ca-CTL group animals were sacrificed 24 or 72 h after *C. albicans *inoculation.

### Ehrlich solid tumor (EST)

This neoplasia, a spontaneous mammary tumor in mice, does not resolve spontaneously. It may evolve to ascitic (EAT) or solid forms depending on the route of inoculation (intraperitoneal or subcutaneous respectively). In this study, the tumor was maintained in the ascitic form in Swiss mice and the cells were collected according to Silva et al. [12]. Tumor cells (1 × 10^7^/100 μl) were inoculated subcutaneously into both the BTM and Ca-BTM groups.

### C. albicans inoculum

*C. albicans *strain FCF 14 was originally obtained from the fungal collection of the School of Odontology, São Paulo State University, São Jose dos Campos, SP, Brazil. The fungi were maintained on Sabouraud medium (Difco Laboratories, Detroit, Michigan, USA), then cultured in the same medium for 24 h at 37°C. The colonies were resuspended and washed twice. Fungal viability was determined by cotton blue staining and the concentration was adjusted to 5 × 10^7 ^viable *C. albicans*/ml [13].

### Collection of the biological material and macrophage culture

Mice were sacrificed using CO_2 _euthanasia, and peritoneal cells were collected by washing the peritoneal cavity with 10 ml ice-cold sterile phosphate buffered saline (PBS), pH 7.4. The suspension was centrifuged and the cells were resuspended in 1 ml RPMI-1640 (Nutricell, Campinas, SP, Brazil) containing 10% heat-inactivated fetal calf serum (Gibco BRL, Grand Island, NY, USA). The cell concentration was adjusted to 2 × 10^6 ^macrophages/ml, as judged by the uptake of 0.02% neutral red. The peritoneal cells were placed in 96-well flat-bottom microtiter plates (Costar, Cambridge, MA, USA) and incubated for 2 h at 37°C and 5% CO_2 _in a humidified chamber to allow macrophages to adhere and spread. Non-adherent cells were removed by washing the wells three times with RPMI, and the remaining adherent cells (>97% macrophages as assessed by morphological examination) were used for experiments. The macrophages were cultured at 37°C, 5% CO_2_, in RPMI-1640 with or without 10 μg/ml LPS (Sigma) as an internal control for macrophage activity (data not shown). After 24 h, the cell-free supernatants were harvested and stored at -70°C pending cytokine analysis. Finally, the peritoneal fluid and liver, spleen and kidney fragments were subjected to microbiological evaluation.

### Direct organ culture

Ten fragments (2 × 2 mm) of liver, spleen and kidney were placed on 15 × 90 mm Sabouraud agar plates at 37°C for 5 days according to a previously-described method [14]. The yeast colonies on the fragments were counted and the results were expressed as the frequency of *Candida*-positive organ fragments.

### Fungal loads in PF

PF (30 μl) was placed on 15 × 90 mm Sabouraud agar plates at 37°C. The yeast colonies were counted on the 5^th ^day and the fungal loads were determined by counting the colony-forming units (CFU).

### Cytokine analysis

TNF-α, IL-12, IFN-γ, IL-10 and IL-4 levels were measured in the cell-free supernatants of the peritoneal cell cultures using a Cytokine Duo-Set Kit (R&D Systems, Minneapolis, USA). Each sample was analyzed in duplicate.

### Statistical analysis

Linear regression analysis was used to determine the correlation between tumor evolution and fungal installation and dissemination. Cytokine production data were analyzed using one-way ANOVA with the Tukey-Kramer post-test [15]. All statistical tests were conducted using GraphPad InStat version 3.0 for Windows (GraphPad Software, San Diego, California, USA) and p < 0.05 was taken as the criterion for statistical significance.

## Results

### Tumor implant

After the EAT cells were introduced in the subcutaneous tissue (EST), the mice developed at the inoculation site a firm, tangible, whitish coloring and relatively movable mass. This tumor showed continuous and progressive growth, leading to the death of the animals at about the 25th day after implantation.

### Fungi installation and dissemination

CTL and BTM animals showed no fungi in any of the samples analyzed or at any time during the experiment.

The frequencies of *C. albicans*-positive animals in the Ca-CTL and Ca-BTM groups are summarized in Table 1. In the Ca-CTL group, intraperitoneal introduction of the fungus resulted in acute systemic candidiasis, with dissemination to the spleen, liver and kidneys. At 72 h, the frequency of *C. albicans*-positive samples decreased, both internally and at the inoculation site, suggesting a tendency towards resolution. In the Ca-BTM group, only one animal after seven days of tumor evolution was *Candida*-positive 24 h pi. At 14 days of tumor evolution, the animals presented a similar picture to the control group. At 21 days, we observed that all the mice showed fungi, but there was exacerbation; the cultivation of tissue samples revealed more viable fungi than in the control group animals [data not shown]. We observed a positive correlation between tumor evolution and the frequency of PF and/or organ colonization after 72 h (r = 0.99; p = 0.04).

**Table 1:** Frequency of *Candida albicans*-positive animals from the Ca-CTL and TBM-Ca groups^a^.

Experimental Group	*Candida *infection hours	Organs^b^			
		
		Peritoneum	Spleen	Kidney	Liver
**Ca-CTL**	**24**	91.7 ± 14.4	80.3 ± 14.4	48.3 ± 44.8	8.3 ± 14.8
	**72**	50.0 ± 0	66.7 ± 28.9	8.3 ± 14.8	8.3 ± 14.8
					
**Ca-TBM** **7^th ^day**	**24**	25.0 ± 0	25.0 ± 0	8.3 ± 14.8	8.3 ± 14.8
	**72**	0	0	8.3 ± 14.8	8.3 ± 14.8
					
**Ca-TBM** **14^th ^day**	**24**	100.0 ± 0	91.7 ± 14.4	33.3 ± 14.4	8.3 ± 14.8
	**72**	50.0 ± 25.0	58.3 ± 28.9	25.0 ± 25.0	25.0 ± 0
					
**Ca-TBM** **21^th ^day**	**24**	100.0 ± 0	66.7 ± 14.4	41.7 ± 52.0	25.0 ± 25.0
	**72**	75.0 ± 0	75.0 ± 25.0	50.0 ± 25.0	58.3 ± 28.9

^a ^The results shown are the average of triplicate experiment. For each experiment, four animals per group were used.

^b ^Data expressed as mean frequency of *Candida*-positive animals (%) ± SD.

### Cytokine determination

In the present study, IL-12 production was only observed in Ca-CTL at 24 h (5.44 ± 1.95 pg/ml).

**Figure 1:**
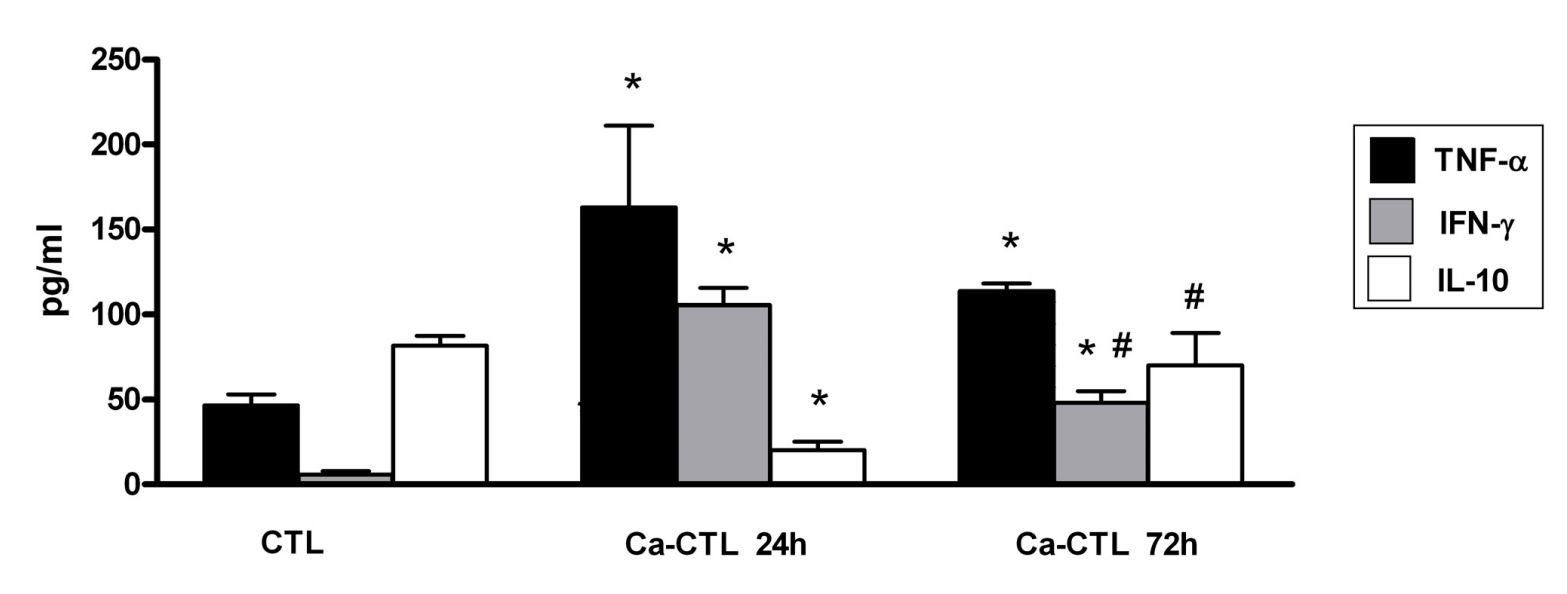
**Release of TNF-α, IFN-γ and IL-10 spontaneously produced by peritoneal macrophages in the Ca-CTL group**. Data are expressed as mean ± SEM. * significantly different from CTL, ^# ^*vs. *Ca-CTL 24 h. P < 0.05; n = 4/group. ANOVA; Tukey's post-test.

**Figure 2:**
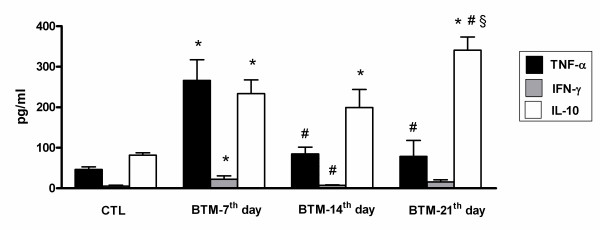
**Release of TNF-α, IFN-γ and IL-10 spontaneously produced by peritoneal macrophages in the TBM group**. Data are expressed as mean ± SEM. * significantly different from CTL, ^# ^*vs. *BTM 7^th ^day; ^§ ^*vs. *TBM 14^th ^day. P < 0.05; n = 4/group. ANOVA; Tukey's post-test.

**Figure 3:**
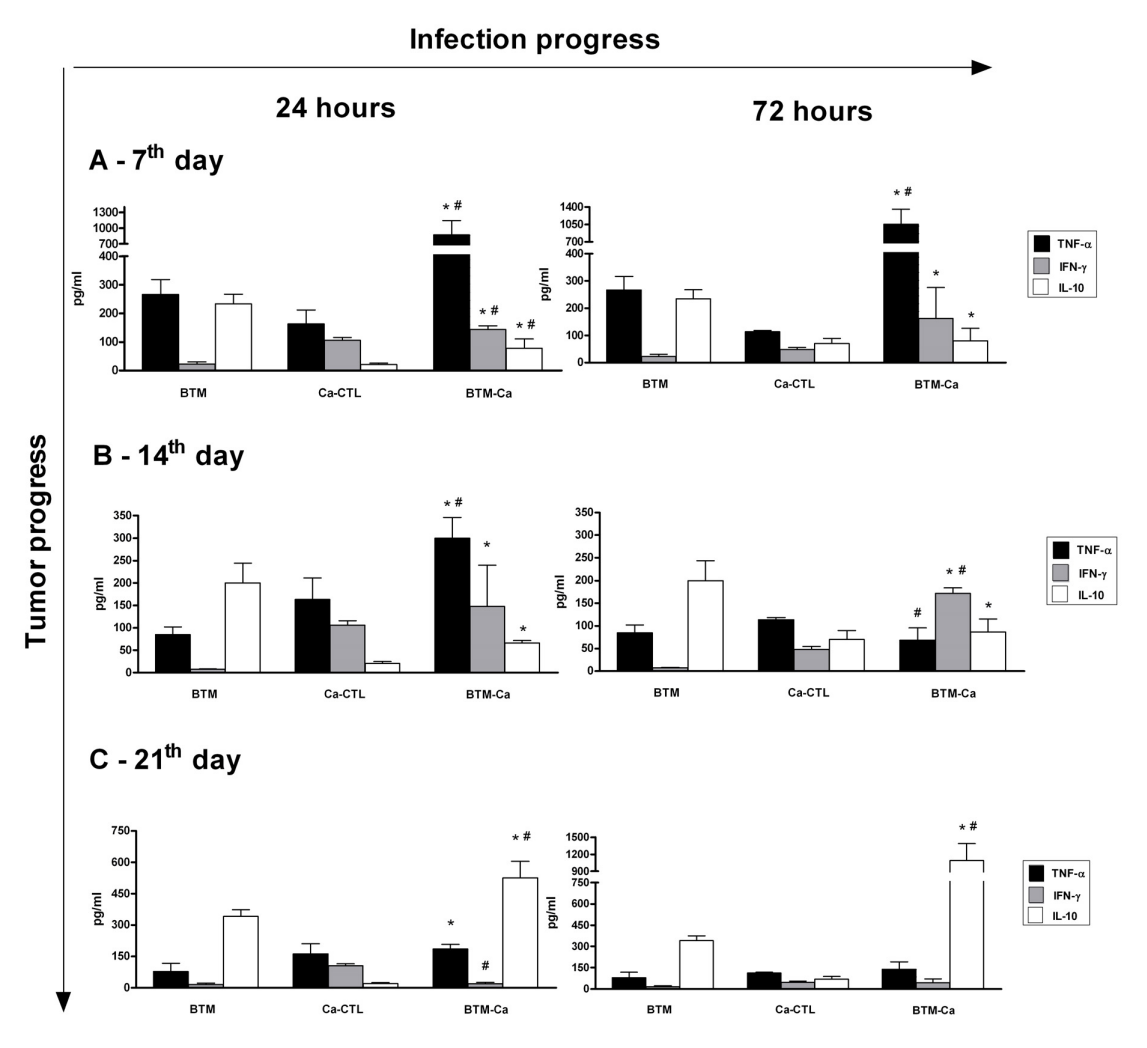
**Release of TNF-α, IFN-γ and IL-10 spontaneously produced by peritoneal macrophages in the Ca-TBM group**. Data are expressed as mean ± SEM. * significantly different from TBM, ^# ^*vs. *Ca-CTL. P < 0.05; n = 4/group. ANOVA; Tukey's post-test.

The level of TNF-α in Ca-CTL increased at 24 h and decreased at 72 h pi (Fig. 1). In BTM, levels of this cytokine increased initially, but decreased with the tumor progression (Fig. 2). A challenge with *C*. *albicans *(Ca-BTM group) did not change this picture: TNF-α increased during the first period of tumor progression, but there was a decrease at 72 h pi after the 14^th ^day (Fig. 3).

To evaluate the immune protective response, we investigated INF-γ production. In our study, macrophages from Ca-CTL group mice showed an increase in IFN-γ production 24 h pi as at 72 h pi (Fig. 1). Although the IFN-γ levels at 72 h remained higher than in the controls, they were significantly lower than those obtained at 24 h (Fig. 1). This datum correlates positively with the fungal concentration in the peritoneal environment, i.e. higher at 24 h than at 72 h. BTM macrophages showed increased IFN-γ production only at the 7^th ^day of tumor progression (Fig. 2). In Ca-BTM, the quantity of IFN-γ measured was higher at the 7^th ^and 14^th ^days of tumor progression (Fig. 3).

To evaluate the anti-inflammatory immune response, we investigated IL-4 and IL-10 production. With rare exceptions, we found no IL-4 in our animals. We verified that the peritoneal macrophages of CTL mice (stimulus-free) could produce IL-10. After inoculation with the fungus, the production of this cytokine decreased at 24 h and returned to base level at 72 h pi (Fig 3A). BTM mice showed a significant increase in IL-10 production throughout tumor progression. In the Ca-BTM group, this cytokine decreased after 7 and 14 days of tumor progression (Fig. 3); at 21 days it increased 24 and 72 h after *Candida *infection.

Taken together, our results show that at the outset of tumor progression, BTM group animals produced large quantities of TNF-α and IFN-γ and little IL-10, and they showed fungal clearance. With the tumor progressed, this picture changed: IL-10 production increased and TNF-α and IFN-γ production decreased, and the animals showed extensive fungal dissemination. Our results also indicate that the tumoral condition suppressed the production of macrophage cytokine IL-12.

## Discussion

Deterioration of immune function and an increased incidence and lethality of infectious diseases caused by *Candida albicans *have been observed in tumor patients [16,17]. Although the mechanisms involved in this association are not totally clear, macrophages are known to play a significant part.

We studied this association in mice with EST, a spontaneous mammary mice tumor of which the development has been related to the activity of the immune system. In a previous study we verified that this tumor significantly decreased the overall resistance of mice to systemic *C. albicans *infection and that this effect was associated with alterations in the oxidative metabolic activity of peritoneal macrophages [18]. As cytokines are deeply involved in both processes (host immunosurveillance against invading pathogens and malignancies), we investigated the possibility that the tumoral condition affects the profile of cytokines released by peritoneal macrophages from tumor-bearing and/or *C. albicans*-infected mice. We studied the expression of IFN-γ and IL-12, the major cytokines produced by macrophages and crucial for a vigorous immune response [19]. We also evaluated the production of IL-10 and IL-4, strong inhibitors of macrophage activation and the inflammatory response [20-23].

We began the study by identifying the cytokines produced by peritoneal macrophages from normal mice (CTL group). We verified that, although incapable of producing IL-12 or IL-4, those cells produced negligible quantities of IFN-γ and significantly more IL-10 than other cytokines. Thus, as observed in rodent strains and also in humans, resident peritoneal macrophage from Swiss mice bear some similarities to the M2 subtype, which typically produce IL-10 [24-26].

Compared with resident peritoneal cells from animals of CTL group, the macrophages from *Candida*-infected mice (Ca-CTL group) produced less IL-10 but copious amounts of IFN-γ and IL-12. The capacity to elaborate high levels of IL-12 and low levels of IL-10 is characteristic of M1 macrophages [27]. M1 macrophages are strong promoters of Th1 immune responses and exert antimicrobial activities, resulting from their ability to secrete reactive nitrogen and oxygen species (NO, peroxynitrite, hydrogen peroxide, superoxide) [19]. In fact, in this microenvironment, the fungal load is obliterated or is markedly reduced. In the absence of fungi, the peritoneal macrophages exhibited the M2 profile again.

Thus, the introduction of *C. albicans *into the peritoneal environment leads to changes of the macrophage phenotype. In agreement with other studies, this reinforces the idea that, even when polarized, Swiss peritoneal macrophages can integrate different signals adequately and can convert their phenotype from M2 to M1 and vice versa.

Tumor progression also provoked alterations that changed the peritoneal macrophage profile: at the outset of the neoplastic process, BTM peritoneal macrophages demonstrated consistent TNF-α production and moderate IFN-γ and IL-10 production. Thus, either they exhibited a functionally mixed population or adopted a rather promiscuous activation state, which could however be ideally suited for diminishing tumor immunosurveillance [28].

With the tumor progressed, the macrophages showed a profile similar to the M2 population. At this time, they became refractory to further stimulation; even when provoked with *C. albicans*, they persisted in exhibiting an immunosuppressive phenotype and could not mount an effective anti-fungal response. These results support the findings of Kuang *et al. *[29], who suggest that new stimuli can fail to stimulate pre-activated cells and subsequently exhausted monocytes/macrophages.

The fact that those phenomena occurred at a site distant from the neoplastic mass indicates that the solid Ehrlich tumor provokes alterations in systemic levels. This may have occurred because of mediators derived from tumor cells and/or of derivative elements in the tumor stroma [29-34]. It is still possible that, along of the progression of the neoplasm, we have collected macrophages originating from a tumor itself. Working with the subcutaneous variety of AK-5, a histiocytic rat tumor, Bhaumik et al. [11] demonstrated hyperactive macrophages accumulated in the peritoneum during tumor progression, and the number of those macrophages decreased drastically when the tumor regressed. Direct labeling of these cells with vital dyes showed that they migrated to and from the tumor site.

From Kuang *et al. *[29], macrophages recruited into the cancer from the circulation may be transiently activated while approaching the stroma surrounding the tumor. When they are in close proximity to the tumor cells, tumor-derived factors would act on them, inducing the M2 phenotype [35]. Our results showed similarities with this assertion, since after seven days of tumor evolution we detected a mixed population of peritoneal macrophages, including pro-inflammatory ones, and with the tumor progression, this population re-acquired a predominantly pro-tumoral M2 subset. As those macrophages have no microbicid profile, they turn the peritoneal cavity into a microenvironment favorable for persistent infection. In fact, with the tumor progression, the animals become more susceptible to *Candida*, as shown by its permanence at the inocolum site, and its dissemination is facilitated.

## Conclusion

Our results showed that at the outset of tumor progression, the mice produced more IFN-γ and TNF-α and less IL-10; they also exhibited fungal clearance. With the tumor progression, this picture changed: IL-10 production increased and IFN-γ and TNF-α release decreased; furthermore, there was extensive fungal dissemination. This picture indicates that solid tumor progression modulates the cytokine profile of peritoneal macrophages, possibly because tumor macrophages continually enter the circulation; a development that may compromise the systemic immune response.

## Abbreviations

TBM: tumor-bearing mice; COBEA: Brazilian College of Animal Experimentation; Ca-TBM: tumor-bearing mice that were inoculated with *C. albicans*; Ca-CTL: mice inoculated only with *C. albicans*; CTL: tumor-free mice and not inoculated with *C. albicans*; EST: Ehrlich solid tumor; EAT: Ehrlich ascetic tumor; PF: peritoneal fluid; CFU: colony-forming units.

## Competing interests

The authors declare that they have no competing interests.

## Authors' contributions

MRC performed the laboratory assays, participated in the sequence alignment, and drafted the manuscript. JV carried out the laboratory assays and performed the statistical analysis. FRVM participated in the immunogical evaluations. MSPA conceived the study, participated in its design and coordination, and helped to draft the manuscript. All authors read and approved the final manuscript.

## Pre-publication history

The pre-publication history for this paper can be accessed here:

http://www.biomedcentral.com/1471-2334/9/98/prepub
